# Abdominal swelling and obstructive uropathy due to hematometrocolpos secondary to imperforate hymen: a case report

**DOI:** 10.11604/pamj.2022.41.18.32582

**Published:** 2022-01-07

**Authors:** Yashvinder Kumar, Priyanka Yadav, Ankit Agarwal

**Affiliations:** 1 Department of Surgery, University College of Medical Sciences, Delhi, India,; 2 Division of Neonatology, Bai Jerbai Wadia Hospital for Children, Mumbai, India,; 3 Department of Pediatrics, Ascension Sacred Heart Hospital, University of Florida, Pensacola, Florida, USA

**Keywords:** Imperforate hymen, hematometrocolpos, abdominal swelling, obstructive uropathy, case report

## Abstract

Imperforate hymen is an uncommon congenital anomaly of the female genital tract and can lead to the collection of blood in the vagina and the uterus. Most patients are not diagnosed until menarche when they present with symptoms such as cyclic abdominal and pelvic pain, constipation, tenesmus, back pain, and difficulties with urination in association with lack of menses. We discuss the case of an adolescent female who presented with the complaint of an increasing abdominal swelling along with the urgency and a sense of incomplete urination. She had not attained menarche. A diagnosis of hematometrocolpos was made based on computed tomography findings. Subsequent perineal examination revealed a bulging imperforate hymen. Hymenotomy was performed with complete resolution of the symptoms. This case highlights the importance of keeping a high index of suspicion for this condition in patients presenting with these symptoms and the importance of appropriate gynecologic examination.

## Introduction

The hymen is a thin membrane of epithelium separating the vaginal lumen from the urogenital sinus. Hymen usually perforates during the perinatal period due to degeneration of the central epithelial cells. Imperforate Hymen (IH) is referred to the persistence of intact hymenal membrane resulting from failed spontaneous rupture. IH is a rare congenital anomaly of the female genital tract with an estimated incidence of about 1 in 2,000 female births [1]. Vaginal outflow obstruction results in the accumulation of blood in the vagina and uterine cavity termed as hematometrocolpos. Most patients are asymptomatic and not diagnosed until menarche. A classic presentation is the lack of menses in a pubescent female with associated cyclic abdominal and pelvic pain [2]. Compression of the rectum, bladder, and ureter can lead to constipation, pain with defecation, or difficulties with urination [2]. A bulging bluish membrane on introital examination suggests an IH. Ultrasonography is the preferred imaging modality to confirm the diagnosis. Magnetic Resonance Imaging (MRI) may be used to further evaluate and assess anatomical abnormalities [3]. Surgical repair of hymen should be performed as soon as the diagnosis is made to prevent the complications. We present a case of increasing abdominal swelling and obstructive uropathy in a 14-year-old female who had not yet started menstruating.

## Patient and observation

**Patient Information:** a 14-year-old girl presented to the surgical outpatient clinic with a history of gradually increasing abdominal swelling over one year. She also reported a recent history of urgency and the feeling of incomplete bladder emptying. She endorsed intermittent cyclic abdominal cramps in the preceding few months and denied having started menses. She denied fever, diarrhea, constipation, vomiting, or weight loss. Her past medical, surgical, and family history was unremarkable. She was seen by multiple health practitioners with no relief in the symptoms despite good compliance with the medical advice.

**Figure 1:**
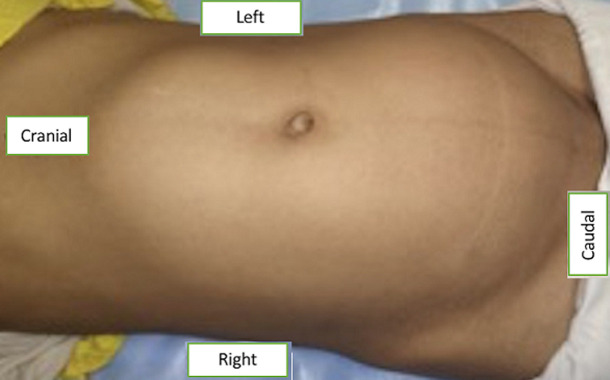
large abdominal mass

**Clinical findings:** the abdominal examination revealed a large non-tender, non-pulsatile palpable mass extending from the pelvis to the xiphisternum (Figure 1). The mass was mainly towards the right side, crossing the midline, and extending to the left. Secondary sexual characteristics were normal for her age (Tanner stage IV). Cardiovascular and respiratory examinations were unremarkable.

**Figure 2:**
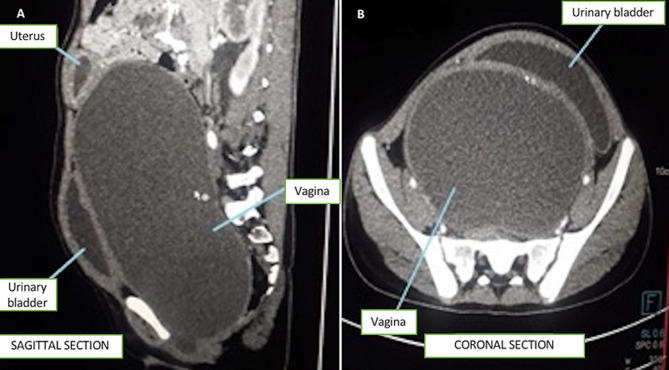
sagittal (A) and coronal (B) computed tomography shows a significantly distended vagina resulting in the compression of the urinary bladder

**Diagnostic assessment:** complete blood count, comprehensive metabolic profile, and urinalysis were unremarkable. The pregnancy test was negative. Transabdominal Ultrasound (TAUS) revealed a large cystic lesion with a thick echoic collection, extending from the epigastric region to the lower pelvis and posterior to the urinary bladder, filling almost the entire abdominal cavity. Computed Tomography (CT) revealed a hypodense, non-enhancing, fluid-filled mass in the vagina measuring 28.5 cm x 11.3 cm x 12 cm with a small collection in the uterine cavity (Figure 2). The lesion was compressing the urinary bladder and distal end of the ureters leading to moderate bilateral hydroureteronephrosis. Subsequent perineal examination revealed a bulging imperforate hymen with bluish discoloration.

**Diagnosis:** a diagnosis of hematometrocolpos was made.

**Figure 3:**
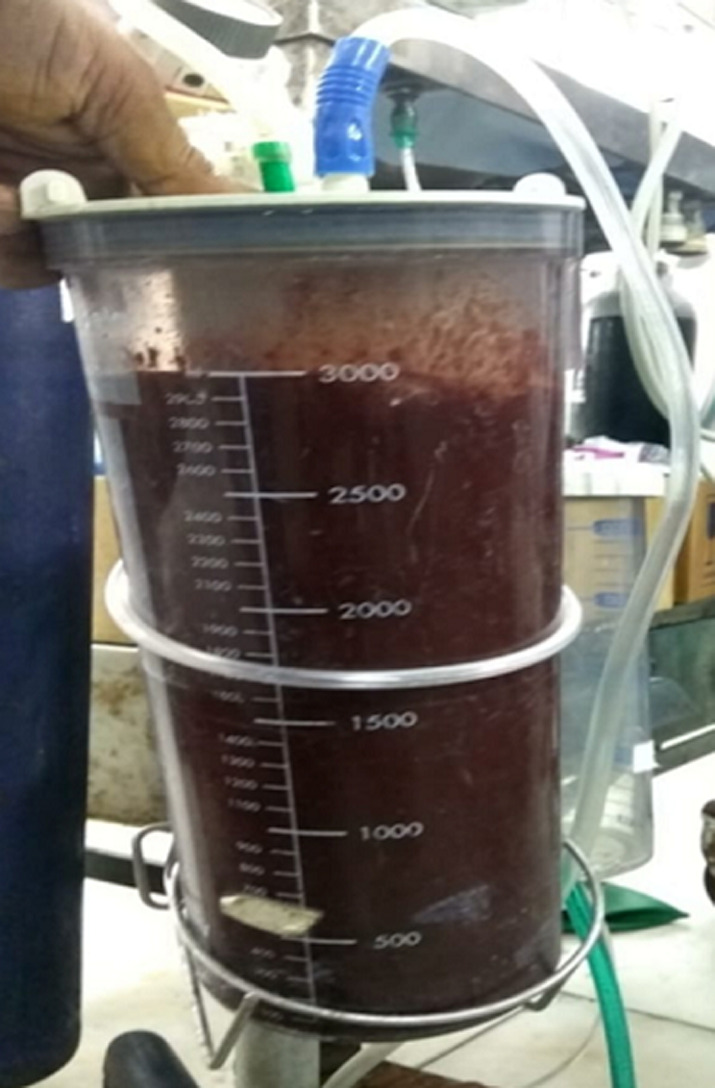
3,000 ml dark-colored blood drained after hymenotomy

**Therapeutic interventions:** the patient and her mother were counselled about the potential risks of surgery including the risk of losing virginity. After taking consent, hymenotomy was performed under anesthesia. Over 3,000 ml of dark-colored menstrual blood was drained (Figure 3).

**Follow-up and outcome of interventions:** the postoperative recovery was uneventful and was discharged from the hospital on the third postoperative day. At the follow-up visit, she was relieved of all the symptoms and had a big smile on her face. Her first menstruation occurred 18 days after the surgery.

**Patient perspective:** “I was so distressed by the illness that I stopped attending school. I underwent multiple evaluations and treatment with no relief before presenting to the surgical outpatient department. I was discharged from the hospital on the third postoperative day, and one week later, I started attending school. At the follow-up, I was relieved of all the symptoms. My menstruation began 18 days after the surgery”.

**Informed consent:** written consent was obtained from the patient´s father to publish images and clinical information relating to the case. The patient was under the age of 18 but had a sufficient understanding of the consent process and its implications.

## Discussion

The hymen is a membrane of tissue and represents the junction of the sinovaginal bulbs with the urogenital sinus. During late fetal development, the centrally located epithelial cells degenerate leading to central perforation of the membrane. Failure of the degeneration results in persistence of the septum which is termed as IH [4]. IH is rare but one of the most common obstructive lesions of the female genital tract, with an estimated incidence rate of 0.05-0.1% [2]. Diagnosis may be made in neonates if a bulging introitus secondary to mucocolpos from vaginal secretions is noted. The mucus is reabsorbed and the child usually remains asymptomatic until menarche if the diagnosis is missed in the neonatal period. Most patients are not diagnosed until menarche when the accumulation of secretions and inability to outpour the menstrual blood results in the formation of a hematocolpos (collection of blood in the vagina) or hematometrocolpos (collection of blood in the vagina and uterine cavity) [5]. In the literature, the frequency of IH with hematocolpos is reported at around 0.14% [6].

At the age of menarche, the females may present with recurrent cyclical and poorly localized abdominal and pelvic pain secondary to distension of the vagina and the uterus by accumulating menstrual blood. The compression of the urethra and bladder can lead to obstructive uropathy symptoms such as urinary retention and urinary frequency as reported in our case [7]. Prolonged or recurrent urinary retention may lead to hydronephrosis and hydroureter [8]. The mass effect can also result in other symptoms like back pain, constipation, and tenesmus. If not treated in time, patients might develop endometriosis by the retrograde blood flow through the uterus and the fallopian tube affecting fertility [9].

The diagnosis can be made clinically by identification of bluish bugling obstruction of the vagina on introital examination. However, low transverse vaginal septum and agenesis of the vagina can result in similar findings. TAUS is usually the first imaging modality. Pelvic MRI may be used to provide a demonstration of the anatomy in complicated cases and the extent of distension of the vagina, uterus, or fallopian tubes [10]. Hematocolpos secondary to IH is managed surgically by making a cruciate or simple incision in the hymen [4]. For patients desiring virginity, hymen preserving surgeries such as annular hymenotomy and simple vertical excision are the options [11,12].

## Conclusion

This case highlights the importance of considering IH as one of the differentials in young females presenting with symptoms such as abdominal swelling, abdominal pain, obstructive urogenital symptoms, and primary amenorrhea. Gynecologic examination and appropriate imaging can lead to early diagnosis and prevent complications like infections, kidney disease, and infertility.
